# Human Behaviour Change Interventions in Animal Care and Interactive Settings: A Review and Framework for Design and Evaluation

**DOI:** 10.3390/ani10122333

**Published:** 2020-12-08

**Authors:** Carmen Glanville, Charles Abraham, Grahame Coleman

**Affiliations:** 1 Animal Welfare Science Centre, Faculty of Veterinary and Agricultural Sciences, University of Melbourne, North Melbourne, VIC 3051, Australia; grahame.coleman@unimelb.edu.au; 2 School of Psychology, Deakin University, Burwood, VIC 3125, Australia

**Keywords:** behaviour change, prevention, intervention, animal welfare, animal handling, animal management, human-animal interaction, human-animal relationship

## Abstract

**Simple Summary:**

In many cases, to improve animal welfare, we must change the behaviour of the people who manage them, care for them, and interact with them. This can be achieved through behaviour change interventions. In this review, we aimed to explore the current state of the behaviour change literature in animal care and interactive settings. We identified significant deficiencies in the design, evaluation, and reporting of these interventions. It was often unclear what behaviours were targeted, how the intervention was designed to work, what was in it, and how it was delivered. Without this information, interventions cannot be rigorously evaluated, built upon by others, or implemented in other settings. Transparent reporting and a structured approach to the design and evaluation of interventions is now required to help the field move forward in a more consistent and robust way. We present such a framework, the “Ten-Task” guide, based on the intervention mapping framework, and demonstrate how its adoption would help progress this field substantially.

**Abstract:**

Behaviour change interventions may be one of the most promising avenues to improve animal welfare. Yet there has been limited systematic research involving them in animal-related settings. We searched three major databases for studies involving an intervention to change interactive or care-related behaviours in any animal-related setting. Forty-seven papers were included in the review and each paper was coded for specific design and evaluation elements. We found a series of limitations in the quality and consistency of intervention design, evaluation, and reporting. Hence, we present a framework, the “Ten-Task” guide, based on the intervention mapping framework, to guide future work in this field. Adopting this structured approach will improve the quality and efficacy of behaviour change interventions for animal welfare and allow for the field to progress in a harmonious way.

## 1. Introduction

We keep and care for animals in a range of different settings from companions in our homes to livestock on farms, research animals in laboratories, and animals in sporting or entertainment contexts. Across these settings, animals are typically dependent on people to provide for their physical and psychological needs and our interactions with animals, in handling and husbandry, have a marked impact on their welfare [1]. Since people’s behaviour as animal owners, stockpersons, animal carers, and even members of the general public (including children) is critical to animal welfare, behaviour change interventions present one of the most promising avenues for improving the lives of animals in our care [1,2].

Numerous animal-related training courses are currently available and there is an increasing interest in research into the effectiveness of behaviour change interventions to improve animal welfare [3]. Research is, nonetheless, much less developed in this field than in other areas, such as human health promotion. For example, ten years ago a review commissioned by the UK’s National Institute of Health and Care Excellence focusing on just six health-related behaviour patterns (healthy eating, physical exercise, smoking, alcohol misuse, sexual risk taking, in young people, and illicit drug use), incorporated 103 systematic reviews [4]. This demonstrates the scale of research into behaviour change interventions designed to promote human health. However, approximately 10–15 years ago, it was recognised that, despite extensive numbers of randomised controlled trials of complex behavioural interventions (representing significant financial investment), the interventions themselves were poorly or inconsistently reported [5]. That is, it was unclear in many cases exactly what had been done, what specific components had worked (or not worked), and how to replicate or apply these findings. As a result, there was a concerted effort to standardise the way in which behaviour change interventions were reported [6,7]. Science as a discipline relies on transparency and replicability for knowledge to be accumulated and extended, without duplicating effort. Thus, with the increasing interest in human behaviour change for animal welfare, by learning from the experiences of other fields and adopting such structured approaches now, we have the opportunity to avoid much wasted time and resources.

Our aim was to review research into behaviour change interventions in animal care or interactive settings, assessing the extent and quality of such research, and to provide recommendations for future developments in the field.

## 2. A Framework for the Design and Evaluation of Behaviour Change Interventions

**Figure 1:**
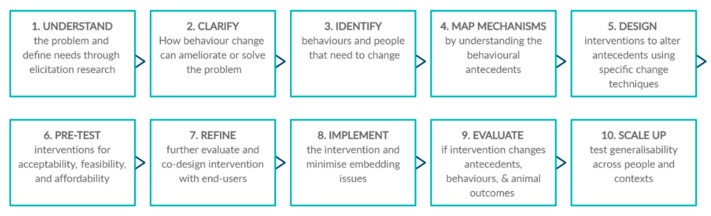
Ten-Task guide to the design and evaluation of behaviour change interventions [9].

The intervention mapping (IM) framework is a detailed guide to best practice in designing and evaluating behaviour change interventions [8]. It has been applied to numerous health-promotion interventions. Abraham and Denford [9] developed a Ten-Task guide based on IM, highlighting key decisions that intervention designers and evaluators need to make to optimise the effectiveness of interventions and the quality of evaluations. This Ten-Task guide is presented in brief in Figure 1. The guide includes understanding the needs of the target population and identifying who needs to change what particular behaviour(s); understanding the mechanisms of change required to generate the desired behaviour change; selecting change techniques likely to produce these change mechanisms; piloting and refining interventions through pre-testing and co-design; understanding implementation; and rigorously evaluating interventions before scaling up to different audiences.

We used this Ten-Task framework to identify aspects of intervention design, theoretical underpinning, content, and evaluation to fully characterise research in the field. We also drew on this framework to identify omissions or weaknesses in current research and thereby identify recommendations to optimise future research into behaviour change interventions in animal-related settings.

## 3. Review of Human Behaviour Change Interventions in Animal Care and Interactive Settings

### 3.1. Study Characteristics

We undertook a structured review of the literature to identify the current state of behaviour change interventions in animal care and interactive settings, particularly the methods used for design and evaluation. The specific elements under review are defined and discussed below.

#### 3.1.1. Design Elements for Review

Change targets: Abraham and Denford [9] highlight that intervention design involves clarifying how behaviour change will solve a problem and precisely identifying the targets of change, including who must change what behaviour patterns in what contexts (tasks 1–3). Specific behaviours and post-motivational planning (e.g., SMART goal setting), as well as prerequisite changes (e.g., knowledge, attitudes, skills) may be targeted in interventions. Without a clear description of what an intervention is aiming to change, it is difficult to evaluate its success.

Theoretical frameworks: Task 4 involves identifying underlying mechanisms of change (also called mechanisms of action), that is, the specific processes through which an intervention is expected to produce a change in the target behaviour, e.g., changing attitudes [10]. There are many psychological theories that describe such mechanisms and consequently, they may also assist in identifying change targets [11]. For example, it may be necessary to change motivation before one can help motivated people change their behaviours. Thus, increased knowledge, changed attitudes, or enhanced skills may become prerequisite change targets. Theories can assist in the identification and understanding of the reciprocal relationships between the target behaviour and relevant antecedents, and interventions are more likely to be effective if they target these mechanisms [12,13]. Understanding what mechanism(s) of change are targeted by an intervention is critical to design and evaluation.

Change techniques: Change techniques may target prerequisites (e.g., attitude change techniques or motivational techniques) or performance of the behaviour itself. While commonly referred to as behaviour change techniques [6], it is critical to map such techniques onto the underlying mechanisms of change and change targets they are designed to alter as highlighted in task 5 [9].

Delivery modes/ procedures: Tasks five and six highlight the importance of understanding how a change technique will be delivered in an intervention. What does the intervention actually ‘look like’? Who delivers it? How many sessions and over what period of time? These are critical factors for replicability and implementation [9].

#### 3.1.2. Evaluation Elements for Review

**Figure 2:**
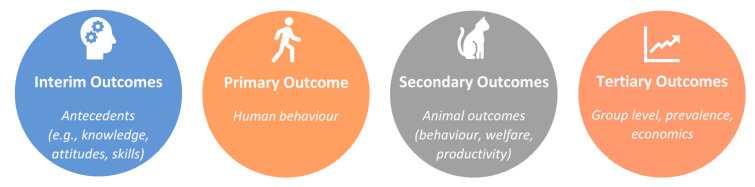
Levels of evaluation.

Evaluation types: The later tasks in the Abraham and Denford [9] guide focus on evaluation. Outcome evaluation is principally concerned with efficacy; does the intervention work and, if so, how well? The primary outcome measure for evaluation of a behaviour change intervention is the targeted behaviour itself [9]. However, additional outcomes may also be important (Figure 2). Secondary outcomes are those resulting from the behaviour targeted at the individual level [9]. In animal care settings, these are likely to include outcomes for the animal, such as improved behaviour, welfare, or productivity. Tertiary outcome evaluation could also be conducted at a group or population level looking at changes in the incidence of the particular issue over time or other associated metrics (e.g., expenditure on regulation/enforcement). Finally, we may also assess changes in interim outcomes such as knowledge, beliefs, attitudes, motivation, or skills, which provide insight into the underpinning mechanisms of change. These evaluations clarify how an intervention worked (or did not work) and are often described as part of process evaluation [9,14]. However, we have included them here in outcome evaluation as many papers in the animal-related domain have used interim measures as a type of outcome without progressing to behavioural measures, thereby not evaluating a process of change. Only by evaluating all levels can true causal pathways between them be demonstrated. However, it is also important to recognise that secondary and tertiary outcomes may be influenced by factors other than human behaviour and these should be taken into account when interpreting evaluations.

It is also important to undertake process evaluations that focus on the process and context of delivery including the fidelity (intervention delivered as planned), dose (quantity of intervention delivered), and reach (number and type of audience reached) [14]. The context of delivery should also be considered as the same intervention, delivered in the same way, may have different efficacy or outcomes in different contexts [14]. Thus, process evaluation can be important for understanding the various factors that will affect the implementation of the intervention in different settings which is especially important for the translation of research into ‘real-world’ programs.

It is also important to know whether the intervention is appropriate for its recipient, that is, its impact on a person, its acceptability, and whether it would be used by the target audience [15]. Feasibility, in terms of the impact an intervention would have on an organisation or provider and the resources required to ensure its successful implementation, should also be considered [15].

Evaluation methods: Depending on the type of evaluation to be conducted a range of different quantitative and qualitative tools may be used [9]. These may include (but are not limited to) interviews, focus groups, questionnaires, behavioural simulations (role-play), behavioural logs or diaries, or epidemiological data.

Tools to evaluate animal-based outcomes are diverse and often species specific. It is beyond the scope of this paper to review welfare assessment methods, though broadly they incorporate tools to assess stress physiology (e.g., cortisol, heart rate variability, oxidative stress), immune function (e.g., immunoglobulin concentrations, incidence of disease), general health and condition (e.g., body condition scoring, lameness scoring), behaviour (e.g., structured behaviour sampling, human and novel object response tests, remote monitoring), affective states (e.g., judgement bias tests), and productivity (e.g., milk production, growth rates, reproductive success) [16].

Experimental design: One of the fundamental aims of intervention studies is to demonstrate that the effect of the intervention on the outcome variables was causal and that the results are generalisable to the population of interest. The most robust experimental method for demonstrating the causal effect of a particular intervention (behaviour change or not) is a randomised controlled trial (RCT) [9,15]. An outline of the various design approaches relevant to behaviour change interventions in animal care settings and their strengths and weaknesses is given in Table 1.

The strongest design is the fully randomised design which depends on genuine random recruitment of participants and random assignment of participants to treatments. These designs do not entail matching of participants, but may be analysed using covariance analysis to partition out variance attributed to known nuisance variables. Next is the randomised block design where participants are matched on a relevant nuisance variable before being randomly assigned to treatment or control groups [17]. Somewhat similar is the randomised preference design where participant preference is determined first before participants are assigned to treatment groups. These designs have several variants [18]. Nested or cluster designs are typically used when participants occupy existing groups such as school classes, workplaces, or communities. In these designs it is important that a sufficient number of groups is recruited to be representative of the population of interest and that the statistical analyses take the nesting into account [19]. Pre–post designs are self-explanatory where before- and after-intervention measures are taken, but with no control group. Observational studies are descriptive exercises to identify possible variables and the interrelationships of interest. Finally, cases studies provide highly detailed information but with no indication of the generalisability of the results.

Timing: Behaviour change necessarily entails the modification of an existing pattern of behaviour, as distinct from learning which usually entails the acquisition of a new piece of knowledge or a new behaviour. Existing behaviour may be quite resistant to change because it is often habitual and may not be associated with a conscious thought process [13]. Short-term changes in behaviour may occur following an intervention while the intervention message is uppermost in the awareness of the participant. However, as the immediacy of the intervention message recedes, habitual behaviour may resume. Therefore, it is important to assess behavioural outcomes over extended periods following the intervention [20]. Typically, this would be several weeks to months following the intervention.

## 4. Methods

### 4.1. Search Strategy and Screening Process

Due to the disparate nature of the current literature, a true systematic review was not possible. The search terms used were (animal* OR pet OR pets OR canine* OR dog* OR feline* OR cat OR cats OR livestock OR farm OR zoo OR laboratory OR abattoir) AND (attitud* OR behav* OR belief* OR motivation* OR knowledge) W/3 (chang* OR interven* OR train* OR educat* OR improve* OR program* OR influen*). Search terms were restricted to the title field to avoid the inclusion of irrelevant medical studies using animal models in their methodology. Three relevant databases were searched in July 2020—Web of Science, Scopus, and Psycinfo. Additional papers were incorporated from the references of those identified through the search procedure. The review process was completed using Covidence systematic review software (Veritas Health Innovation, Melbourne, Australia). After duplicates were removed, the titles and abstracts of all papers were screened for relevance. Papers clearly not meeting the inclusion criteria (outlined in Section 4.2) or belonging to a different discipline (e.g., medicine) were excluded. Where further information was required to determine whether the inclusion criteria were met, the papers were progressed to a full-text assessment. Each step was conducted by the first author (CG). As this is not intended to be a systematic review and the selection criteria were based on objective measures, a single reviewer was deemed adequate.

### 4.2. Inclusion/Exclusion Criteria

Primary experimental research studies published in peer-reviewed journals were considered for inclusion. To be included, studies had to: (1) address some form of animal care, husbandry, or interactive behaviour; studies focused on conservation, consumer choices, and human health were excluded, and (2) incorporate an intervention to change either behaviour or behavioural antecedents (e.g., attitudes). All types of research design were included as this was an element of the review and no criteria were set for research quality.

## 5. Results

**Figure 3:**
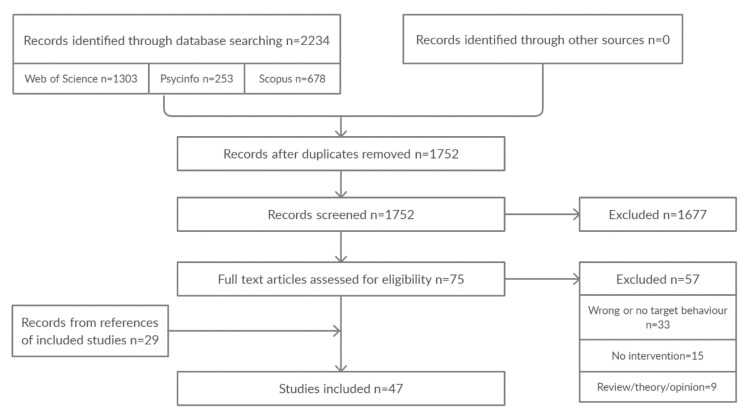
Screening process for structured review.

Figure 3 illustrates the review process. A total of 47 studies were found to meet the inclusion criteria.

### 5.1. Coding of Design and Evaluation Elements

Each included study was reviewed in detail to code the various design and evaluation elements of interest to an Excel spreadsheet. Where required, supplementary materials were consulted. The study design and evaluation elements for the 47 included studies are summarised in Appendix A—Table A1 (research design elements), Table A2 (intervention content elements), and Table A3 (evaluation elements). Currently, no taxonomy or standardised reporting language is used for behaviour change techniques in the animal care field. Hence, we mapped the intervention content onto types of change techniques identified in previous analyses within the health domain. We drew on the 26 categories of change techniques identified by Abraham and Michie [6] (AM1—AM26). These categories were used as they are helpfully mapped onto the relevant theoretical frameworks so that change technique types are directly linked to the mechanism of change they are designed to alter. Where techniques could not be mapped onto these 26 categories, we drew upon the 123 categories listed by Knittle et al. [21] (K1–K123). This list of technique types, that can be used to enhance motivation and translate motivation into action, is the most comprehensive published to date. In some cases, we developed change technique categories that were not defined in earlier lists, so beginning the process of developing change technique categories specifically tailored to animal-related behaviour change interventions.

### 5.2. General Observations

Through the screening process, it became clear that there is a relatively large number of studies examining attitudes and other antecedents of behaviour, some with recommendations for interventions, but comparatively few that progress to fully designing and trialing an intervention. This is likely because intervention trials require significantly more resources than attitudinal studies, which often use surveys. However, it does highlight the need to move beyond attitudinal research to test theories of change in this field and to develop effective behaviour change interventions.

A substantial number of general animal welfare education or humane education programs were found. Those that concerned animals that the target audience were not in direct contact with (e.g., general public or school children with farm animals or animals in research) and did not explicitly aim to address an animal care, husbandry, or interactive behaviour were excluded, e.g., [22,23,24,25,26]. However, despite being outside of the scope of the structured review, some of these studies utilised novel techniques that may be promising for integration into more specific behaviour change intervention trials in the future. For example, Hazel et al. [27] demonstrated that teaching students to clicker-train chickens can improve attitudes towards animals and belief in their cognitive capacities; Helton and Helton [28] found that presenting information about the cognitive capacities of animals increased ‘pro-animal attitudes’; MacKay et al. [29] used a Massive Open Online Course (MOOC) to reach a very large audience (33,501 people enrolled and 5501 completed the 5 week course); Małecki et al. [30] used literary fiction to improve attitudes towards animals; and Tsai and Kaufman [31] demonstrated that a virtual pet ‘Nintendogs’ can increase children’s empathy and humane attitudes. What is now needed is for these techniques to be applied to specific behavioural targets and assessed for their ability to change behaviour.

Two papers that focused on increasing human physical activity for human health benefits utilised dog walking as a strategy to achieve this [32,33]. As these studies were situated within the human health promotion field, the design and evaluation of these studies more closely aligned with the strict design, evaluation, and reporting guidelines of the health psychology field. They used randomised controlled trials and theoretical frameworks, clearly reporting their behavioural targets and behaviour change techniques. However, these were excluded from the structured review as the aims and intended outcomes were not animal-based, despite potentially having positive side-effects for the animals.

### 5.3. Animal Care or Interactive Settings

The interventions found covered a wide range of topics across several settings including animal production (livestock), animals in research, and companion animals (Table A1). One key area in which humans use and care for animals that no behaviour change interventions were found is the use of animals for entertainment. Interventions in the animal production setting mainly involved pig and cattle farming, with two papers on working donkeys [34,35], and single papers on sheep [36], mink [37], and abattoir handling [38]. Two studies focused on the use of animals in research, associated animal care, and the 3 Rs (Replacement, Reduction, Refinement) [39,40]. Companion animal-related studies were primarily conducted with children in the school environment, focusing on dog bite prevention and related ‘responsible pet ownership’ practices. Interventions with adults included two studies on cat containment and overpopulation [41,42], two studies on dog obesity [43,44], general cat and dog management in the community [45], rabies prevention [46,47], and dog behaviour and the human–animal relationship [48].

## 6. Discussion of Design and Evaluation Elements

### 6.1. Intervention Targets

#### 6.1.1. Target Behaviours

A clear understanding of the desired behavioural outcome of an intervention is not only important for designing and implementing the intervention, but also for evaluation and replication; did the intervention bring about change in the appropriate behaviours? In the present review, target behaviour(s) were often poorly defined and at times not at all (Table A2). Broad categories of behaviour were often reported such as ‘responsible dog ownership’, ‘safe behaviour’, and ‘improved handling’, with little explanation of what specific behaviours were involved. For example, Coleman et al. [49] aimed to reduce ‘aversive behaviours utilised by stockpeople in moving breeding pigs’ (p. 15), though what constituted aversive behaviours was not defined (although it was defined in earlier research [50]). Rayner et al. [51] aimed to improve veterinarians’ knowledge about and attitudes towards animal welfare and euthanasia. However, no target behaviours were defined or evaluated and it was difficult to deduce what specific behaviours would be expected to change. Sometimes behaviours were only broadly defined in the methodology, but able to be deduced from the evaluation measurements used. For example, Utami et al. [47] aimed for owners to ‘provide better care [for] their dogs’ (p. 2), and for dogs to be presented for vaccination (p. 3). Evaluation measures included self-reported confinement practices and animal outcomes such as body condition scores, skin problems, and sterilisation status. Therefore, it can be deduced that the intervention was designed to have owners (1) present their dog for vaccination, (2) confine their dogs to their property (but not tethered or caged), (3) feed their dogs an appropriate quality and amount of food, (4) provide preventative or reactive treatment for skin conditions, and (5) have their dogs sterilised. Clearly articulating these behavioural targets would provide readers with a better understanding of the project aims and how these were matched to evaluation measures. Additionally, the behaviour associated with each welfare indicator needs to be explicitly assessed.

There were also examples of well-defined behavioural targets. Grant et al. [36] very clearly defined behavioural targets as this was actually part of their intervention; promoting six steps that farmers could take to implement best-practice treatment of foot rot in sheep. These six steps were based on elicitation research including focus groups, interviews, and a telephone survey with both ‘experts’ and farmers, identifying barriers and motivators to treating lame sheep. Coleman et al. [20] provided a clear description of ‘correct’ behavioural responses to an accompanied and unaccompanied dog that the intervention was aiming to teach children: standing still with hands by side, looking away from the dog, and slowly backing away, hands at side; asking the owner whether they could pat the dog, holding out the back of their hand for the dog to sniff, patting the dog under the chin, and moving away from it if it appears unhappy (p. 275). Explicit descriptions such as these are essential for accurate evaluation of the effectiveness of an intervention.

#### 6.1.2. Intervention Content

The content of interventions may target a range of different behavioural antecedents (e.g., knowledge, attitudes, skills, resources) or behaviours directly. These targets should be based on elicitation research identifying those modifiable factors most likely to impact the behaviour or bring about change. General knowledge was the most commonly targeted behavioural antecedent with many studies predicated on the assumption that if people were more informed about the issue, they would perform the desired behaviours, e.g., [52,53]. Stringer et al. [34] and Stringer et al. [35] targeted knowledge exclusively and did not discuss human behaviour change, but instead asserted that ‘One approach to decrease the prevalence of wounds is through education of donkey users.’ (p. 91). This would undoubtably involve a change in the owner’s behaviour but this was not recognised or discussed. Behaviours regarding wound treatment and to some extent, prevention, could be deduced from the learning objectives, but clear identification and reporting of the desired behaviours is necessary.

While some studies had conducted elicitation research which identified knowledge to be lacking and therefore a potentially influential factor, others did not, which could result in wasted time and resources on an intervention that is not targeting the relevant factors. For example, Chilundo et al. [54] demonstrated significant improvements in farmer knowledge about confinement practices for pigs, yet no changes in confinement practices were observed. Additionally, at baseline, while more than 40% of participants knew how to construct and maintain a model pig pen, none had fully implemented this model, suggesting there were other contributing factors other than knowledge. However, they did demonstrate improvements in this knowledge after the intervention (85.7% knew how to construct and maintain the model pen) and 44.9% of trained participants adopted the model pen design. However, given the baseline data, it does beg the question as to whether the improvement in model adoption was indeed a result of improved knowledge or whether the intervention influenced other factors such as perceived importance of model adoption or perceived social pressure, as the program was conducted in a community setting and had ‘remarkable spillover’ (p. 1447) to other untrained villagers. Machila et al. [53] provides an example in which the intervention study was actually based on a preliminary survey [55] that identified farmers’ knowledge of the causes and indicators of bovine trypanosomiasis to be lacking and concluded that this was the reason for their poor disease control practices. However, only knowledge and self-reported behaviours were measured in the survey; no other potential drivers were considered (e.g., attitudes, values, resources). Additionally, they did not use any predictive (regression) or even correlational analyses to link the two factors; only descriptive analyses were performed. These examples highlight that while knowledge may be a prerequisite, it is also important to consider and appropriately evaluate other antecedents in elicitation research. Drawing on theoretical frameworks of behaviour will assist in taking this more comprehensive approach.

While behavioural models often appear unidirectional with antecedents leading progressively towards the final outcome of behaviour, in reality there are reciprocal relationships or feedback mechanisms that should be considered [56]. That is, while antecedents like attitudes may drive behaviour, our behaviour also has a role in shaping our attitudes. This is particularly true if our behaviour is not aligned with our attitudes and it is often easier to change (unconsciously) our attitudes than to change our behaviour, particularly if it is habitual. Recognising this relationship several studies employed a ‘cognitive–behavioural’ approach, targeting both these cognitive antecedents and the behaviour directly [49,50,57]. In doing so, it was concluded that a more enduring behavioural change would be achieved, effectively inducing a self-reinforcing system.

In a similar vein, there is also a reciprocal relationship between human behaviour and animal behaviour; human behaviour impacts animals and their behaviour influences human behaviour [58]. For example, a stockperson may consider pigs to be difficult to handle and therefore use more force and aversive behaviours when doing so. Consequently, the pigs fear the stockperson and this fear translates to behaviours that make them difficult to handle, thus reinforcing the stockperson’s beliefs and behaviours. Clark and Boyer [48] directly targeted both human and dog behaviour through obedience training and behavioural counselling. This is likely a particularly useful strategy for companion animals considering the general acceptance of training them and practical ease in doing so. It would be practically quite difficult to systematically train production animals, though this is an interesting prospect and likely inadvertently occurring, e.g., training dairy cows to come when called for milking. The Clark and Boyer [48] study was the only one found to directly target animal behaviour and given the importance of this relationship this method should receive more investigation.

#### 6.1.3. Target Audience

Another major consideration is the intervention’s target audience; who needs to change and what is the best way to reach them? Most interventions use a direct approach, delivering the intervention directly to their target audience. However, several studies utilised an indirect approach for various reason (discussed further in 6.4 delivery modes).

Another consideration is whether the intervention is appropriate for the target audience. In this regard, Morrongiello et al. [59] investigated whether the dog bite prevention interactive CD “The Blue Dog”, which had shown positive impacts on children’s knowledge in other studies [60,61], and was originally developed with the aim of influencing both child and parent behaviour, would indeed have a positive impact on parent supervisory behaviour. They found that it did not, highlighting that what works for one target audience may not for another. Consequently, the target audience must be carefully considered and ultimately consulted in the design process. One way to do this is the process of co-design (i.e., including end users and relevant stakeholders in the design process [62]) which is an important element of the Ten-Task guide [9].

Co-design was used well in several of the studies reviewed. Machila et al. [53] involved the target audience in each step of the process. Prior to developing the intervention materials (posters and leaflets) they conducted focus groups with farmers to discuss the proposed contents, then after they had developed prototypes these were pre-tested with farmers (focus groups) and subsequent revisions were made. They also discussed with farmers dissemination formats and channels, and used a preference ranking system to determine the best approach. Deray et al. [63] conducted a planning workshop with 27 head teachers to develop an implementation plan and ‘strategies to institutionalise the rabies curriculum integration’ (p. 6). Selected senior teachers drafted the intervention manual specifically for their area (El Nido), which was then pre-tested with other teachers for content and practicality of use.

### 6.2. Theoretical Frameworks

Very few studies reported using a theoretical framework to identify or articulate their mechanisms of change. The most comprehensive and integrated use of theory was McDonald and Clements [41] research into cat overpopulation. In addressing this complex problem they used the Michie et al. [64] COM-B model and associated Behaviour Change Wheel. A separate paper was dedicated to applying these models to the chosen problem, identifying the drivers of the chosen behaviours, and proposing appropriate intervention functions and techniques for each [65]. This is admirable and this close consideration of underlying change processes is critical.

Two studies utilised the Theory of Change approach [47,66] which is characterised by first defining a long-term goal before mapping backwards the steps required to reach that goal. The application of this theory by Utami et al. [47] demonstrated the proposed relationships between target owner behaviours (‘guardians take good care of dogs’ and ‘dogs are presented for vaccination’ p. 3) and their ultimate goal (rabies control), but did not incorporate antecedents of the target behaviours. Therefore, they did not articulate the mechanism of change, i.e., how the intervention would change behaviour. Baatz et al. [66] utilised the same Theory of Change approach, but incorporated attitude and knowledge change into their change model. In this way, they clearly articulated their hypothesised change mechanism; the two interventions would increase specific areas of knowledge which would in turn change specific attitudes and behaviours, leading to their ultimate goal that ‘every dog can enjoy a happy life free from the threat of unnecessary destruction’ (p. 3).

Bright and Hadden [67] used the Dick and Carey [68] Model of Instructional Design which aims to train specific behavioural ‘domains’—intellectual (knowledge), attitudinal, verbal (knowledge), psychomotor (skills). These domains are reflective of other leading psychological theories and provide a comprehensive approach to behavioural change. The authors clearly described the learning objectives for each of these domains, though how this was achieved and evaluated was less clear.

The lack of integration of psychological theory has seemingly resulted in many interventions simply adopting the premise that if people know more, they will change their behaviour. While in some cases this may be true, it is likely that there are many more interacting elements such as motivation, skills, and resources that drive behaviour and behavioural change. Additionally, the relevant factors and their relationships with each other are likely to vary across contexts and with different target behaviours; for some behaviours, behavioural skills may have a direct influence on behaviour, while attitudes may mediate this effect for others. It is important that elicitation research is conducted to develop a domain-specific theoretical framework for the intervention. Drawing on existing psychological theories will assist interventionists in identifying the relevant factors and addressing them in more effective ways than simply providing ‘education’.

### 6.3. Change Mechanisms and Change Techniques

We examined mechanisms of change identified in intervention evaluations and the type of change techniques included in interventions. Unfortunately, this was challenging because interventions were often poorly described making identification of precise content difficult. For example, many school-based programs only provided brief summaries of lesson topics or broad descriptions of activities such as games, discussions, and hands-on activities, e.g., [69,70,71]. Moreover, some reports did not describe the intervention content at all, e.g., [39,40,45,63,72]. This is problematic scientifically because as Nicoll et al. [70] note, “we do not know what exactly it is [in the intervention] that is effective.” (p. 55). In such cases an intervention cannot be replicated, an effective intervention cannot be adopted with fidelity, and no advance is made in understanding the processes of change involved. If a science of intervention development and evaluation is to be established in relation to the care and handling of animals, then more precise reporting is required [73]. Most journals now allow the publication of supplementary documents facilitating the full reporting of intervention manuals and some journals now *require* submission of manuals before publication of intervention evaluations [74,75]. This practice should be more widely adopted in animal-related fields.

Many change mechanisms were targeted in the interventions described by the 47 papers. In some cases, these were clearly identified (as recommended by Abraham and Denford [9]), but in others they had to be derived from less explicit descriptions. We used a range of theoretical frameworks onto which these change mechanisms could be mapped. The Information Motivation Behavioural skills model (IMB) [76], highlights the importance of pre-existing knowledge and development of skills. Both the Theory of Planned Behaviour (TPB) [77] and Social Cognitive Theory (SCogT) [78] identify precursors of motivation including attitudes, normative beliefs, and self-efficacy, all of which may become prerequisite targets and the foci of evaluations. Social Comparison Theory (SComT) [79] emphasises the importance of comparing ourselves to others, thereby engaging in vicarious learning (see too SCogT) and altering normative beliefs (see too TPB and SCogT). Control Theory (CT) [80] emphasises the importance of goal setting, self-monitoring, and goal review in translating motivation into action. The Context, Executive, and Operational Systems theory (CEOS) [81] distinguishes between habitual and non-habitual action sequences, highlighting the importance of making and altering habits in behaviour change. Finally, learning theory, including specification of operant conditioning (OC, [82]), emphasises how reinforcement of behaviour over time generates repetition and routinisation that is foundational to habit formation.

To undertake these mappings onto underlying change mechanisms and the types of change techniques, we first reviewed the 47 intervention descriptions provided in the reports that met our inclusion criteria. These examples were then categorised in terms of the mechanism of change identified by the theoretical frameworks we had selected and the categories of change we could identify. The data are presented in Table 2 with specific examples from studies, the associated targeted change mechanisms, and deployed change technique types.

Many techniques focused on the identification of recommended behaviour and/or instruction on how to perform the recommended behaviour. Demonstration of the desired behaviour was the most commonly identified technique typically involving role play and modelling. This technique draws on Social Cognitive Theory [78], using social learning to improve behavioural skills and self-efficacy (personal belief in ability to perform the behaviour). Instruction on how to perform the behaviour was also commonly used, particularly teaching participants how to interpret animal body language and how to interact with them appropriately. This was often a key component of dog bite prevention interventions, teaching children how to interpret dog body language and judge risky situations. Consequently, it is clear that even though Social Cognitive Theory was not identified or mentioned by any of the papers reviewed, many techniques reflect its underlying principles and mechanisms related to vicarious learning and self-efficacy enhancement.

Some commonly used techniques related to execution and behavioural regulation; key concepts of Control Theory. These included the facilitation or guiding of participants in problem solving, goal setting, action planning, behavioural practice, and receiving feedback on performance or outcomes. Roetman et al. [42] used a particularly novel approach to provide cat owners with feedback on the outcomes of their cat management behaviour, specifically letting cats out to roam, by tracking pet cats with GPS collars. This strategy was based on previous research suggesting that one of the barriers to cat containment was that owners lacked an accurate understanding of how far and where pet cats roam. Interestingly, the intervention did not make any explicit requests for participants to contain their cats, but did find a significant increase in self-reported containment behaviour as a result of the intervention. Several studies also incorporated some form of follow up (e.g., newsletters and visits) or environmental cues (e.g., posters in workplace) to reiterate the intervention’s messages and prompt behaviour. However, it was often unclear what was contained in these follow-up materials and they could well have incorporated additional, undescribed change techniques.

Taking a more cognitive approach, providing information about the consequences of the behaviour and information about behavioural antecedents were also common strategies. While these techniques involve the provision of information, they are much more specific than basic awareness, providing specific types of information to facilitate attitudinal change. Attitudes towards a behaviour are informed by our beliefs about the consequences of that behaviour, how likely those consequences are, and our evaluation of whether the consequences align with our personal goals (values) [77]. Hence, providing people with information about the consequences of behaviours and how various antecedents (e.g., attitudes or beliefs) impact on those behaviours are useful strategies to influence attitudes. These techniques were applied in multiple ways. Coleman et al. [49] presented participants with information about the consequences of aversive handling techniques on the ease of handling and pig productivity. This included the presentation of data from previous studies demonstrating the link between stockperson attitudes and behaviours, and pig behaviour and productivity. They also showed video footage of appropriate and inappropriate handling behaviours and the associated behavioural responses of pigs. In this way, they showed the consequences of the behaviours, but also demonstrated the appropriate behaviour. Grant et al. [36] also presented information about behavioural consequences, but they took this one step further and also investigated the framing of such messages; whether a positively framed message (production gains) would have different outcomes than a negatively framed message (production losses).

Also important to consider are those techniques used in other fields of behaviour change that were not identified in the present review or used infrequently. Particularly, the use of cognitive and motivational techniques could be improved. For example, social norms are an important motivator for many behaviours and yet only one study utilised change techniques that facilitate social comparison and reflection on norms [38]. Other examples of cognitive techniques that were not identified at all include motivational interviewing, reflection on one’s values and self-identity and how they align with the behaviour, or self-persuasion. Additionally, few interventions considered habits or emotions, which are also complex contributors to the performance and maintenance of behaviour [81]. Knittle et al. [21] list 123 change techniques that have been used and tested (to varying degrees) in other fields. Hence, we would urge intervention designers to consider the full range of available techniques and begin to incorporate more complex approaches.

Overall, while there were some good examples of intervention descriptions enabling coding of specific techniques and mechanisms, reporting was generally poor. Put simply, if we do not know what is in an intervention, there is no way that it can be implemented by others or built upon to advance the field. Additionally, there is a wide range of change techniques and mechanisms currently unused in this field that could increase the efficacy of interventions.

### 6.4. Types of Interventions and Delivery Modes

Where change techniques and mechanisms describe the active content of the intervention, this can be delivered in a variety of ways. The types of interventions and delivery methods identified in the current review were highly varied, from a single 30 min education session to a 4 year multifaceted community outreach program. However, the majority involved some sort of ‘education session’.

School-based interventions were either a ‘guest visit’ by an outside instructor or integrated into the curriculum, with lessons being delivered by the students’ regular teachers. Guest visits were often accompanied by a temperament tested dog for demonstrations and behavioural rehearsal [20,70,90]. Arbour et al. [84] highlighted ethical issues related to using animals (demonstration dogs) in such education programs and instead used a literature-only program, which resulted in increased empathy, but no significant change in behaviour. Nicoll et al. [70] compared an animal-assisted program to a literature-only approach, finding that the animal-assisted sessions improved self-reported empathy but not self-reported behaviours, while the literature-only approach did neither. Consequently, if the ethical issues related to using live animals in education are to be avoided, more effective animal-free programs need to be developed and evaluated.

Aside from simple education sessions, there were several different intervention approaches that warrant highlighting. Utami et al. [47] used a multifaceted community-based program incorporating both education and direct support (free veterinary services and health days). They trained members of the community to go door to door within their own village, surveying, documenting, and generally promoting the program. This approach is likely to improve acceptance and community ownership of the intervention, particularly among communities or target audiences that may be resistant to outside instruction. Several other studies utilised this approach of indirect dissemination; Descovich et al. [38] used a ‘train the trainer’ approach whereby they delivered training (and paid for the associated enrolment and travel costs) to abattoir staff at managerial levels who were then expected to train at least 20 other staff within their facilities; Machila et al. [53] educated school children (in addition to village elders, animal health centres, and Agrovet shops) about bovine trypanosomiasis and associated drug use with the intention that they would take the message home to their households; and Karimuribo et al. [83] delivered training to extension officers, animal health officers, and key community individuals, while their primary target was farmers. This method of indirect dissemination is likely to be an effective strategy to maximise reach and may also assist with acceptability. However, there is potential for dilution effects and reduced fidelity in the content delivery and as such, this needs to be evaluated.

Several studies compared the efficacy of different types of interventions. Stringer et al. [35] and Stringer et al. [34] compared three delivery modes; a diagrammatic handout, a village meeting, and a radio drama. In both trials, (2011 and 2018) they found that the handout and village meeting were more effective than the radio program. Similarly, Grant et al. [36] compared a postal intervention, group-based intervention, and a one-on-one intervention. Perhaps unsurprisingly, they found that a reduction in sheep lameness (desired outcome) was greatest for the one-on-one group and lowest for the postal group. This highlights a common dilemma for interventionists; a one-on-one, personalised approach may be more effective but is more resource-intensive and reaches fewer individuals. Conversely, large scale generic messaging may reach a much larger audience, but the degree of change may be much less. In some cases, mass education campaigns may be used before local or individual interventions, in order to increase knowledge and begin to challenge attitudes before investment in more intensive behaviour change interventions.

Another distinct intervention style was the use of ‘stable schools’ or ‘farmer field schools’ [37,86,87,88]. These involve small groups of farmers who meet regularly to discuss specific problems they are encountering on their farm and facilitate knowledge exchange to collectively find solutions to the presented problem. The groups are assisted by a facilitator for administrative purposes (minutes, scheduling) but there is no input from any external ‘experts’ in terms of content or advice. This approach is based on self-determination theory and participatory approaches to empower participants through common learning. While these types of interventions are generally highly desirable and acceptable to participants and would minimise embedding issues associated with resistance to outsiders, their efficacy in driving positive change in situations where the knowledge-base or attitudes of participants is not optimal, would need to be further examined. Indeed, such interventions could have negative consequences, for example if they were conducted with dog owners (whose knowledge and attitudes are highly variable) and inappropriate training or management techniques were recommended and adopted by participants.

In contrast to these more participatory/common-learning approaches, several studies utilised a ‘credible source’, where information or a persuasive message is presented by an influential person (e.g., relevant experts, celebrities, organisations, or people with lived experience). An interesting example of this was Shen et al. [85] who used real-life testimonials of paediatric dog bite incidents read by four adult actors. They specifically chose to use adults to present the testimonials as opposed to the children themselves as they had established that in China (the study country) children ‘respect and admire adult authorities’ (p. 5) and would be more likely to take the messages seriously. Another example of this approach involved incorporating quotes with a photograph of a specialist sheep veterinarian and a sheep farmer in a leaflet about the management of sheep foot rot [36]. It should be noted that some taxonomies of behaviour change techniques define the use of a credible source as a change technique. However, it is in fact a delivery mode feature because any message can be presented by a more or less credible source and credibility is critical to message acceptance.

### 6.5. Research Design

As mentioned previously, the randomised controlled trial (RCT) is the most robust experimental method to demonstrate causality of an intervention. Of the 47 intervention studies, 16 were considered to meet the criteria of a RCT (separate control group and random allocation of participants to treatments). Shen et al. [85] provides a good example of the RCT including randomisation within classrooms to reduce clustering effects and clear reporting using the Consolidated Standards of Reporting Trials (CONSORT) flow diagram [94].

However, few of the RCTs employed genuine random sampling which can result in sampling bias [95]. In animal care-related studies this largely arises from the need to use volunteers who may not be representative of the target audience for a range or reasons. For example, people who volunteer to be involved in an animal welfare-related study may be generally more committed to animal welfare. Consequently, they may not include those who have a record of poor welfare, or due to various social barriers, may not include individuals of lower socio-economic status. An example of this is Byers et al. [43] who recruited participants for their dog obesity intervention through a tertiary care veterinary referral hospital. They provided a brochure to clinic attendees that explained the study and then those that expressed an interest in participating were included. Therefore, the sample may be biased towards (a) people who place enough value on their dog’s health to take them to a specialist centre, (b) have the money to do so, and (c) given participants volunteered after reading about the purpose of the study (dog weight loss), they may already have some of the motivational pre-conditions required to undertake the desired behaviours. This was a limitation recognised by the authors. Another example is Grant et al. [36] where even though they invited a random selection of farmers to participate in the group intervention (n = 400) only 78 farmers actually volunteered to participate. Consequently, care needs to be taken in generalising the results of such studies if volunteering reflects a personal characteristic that is likely to affect the dependent variable. Such studies can be generalised to the extent that the intervention can work in volunteers, but it may be uncertain whether it would work in non-volunteers. One way to address this is to investigate *why* people did not volunteer to determine whether there is some form of systematic bias. This was utilised in several of the studies reviewed here, e.g., [34,54]. Alternatively, compiling data about the non-volunteer group may be helpful, e.g., whether they match national census data on socio-economic characteristics such as age, gender, education, or employment status.

Another design that attempts to deal with selection biases is the fully randomised preference design [18] which ensures that participants are informed of and treatment groups matched on participants’ outcome preferences. There are various strategies within such designs to reduce biases that we will not discuss in detail here, but which are covered by Ainsworth et al. [18]. This approach was not utilised in any of the included studies.

A further source of bias can arise if a double-blind method is not used. Tarquinio et al. [95] argued that a way of dealing with this is to use such designs as cluster randomised design trials where social units such as school classes, farms, or communities comprise the experimental units. This was used relatively frequently in the papers reviewed here, particularly with schools and villages.

Community interventions can be difficult to control. The best option is to match discrete target areas (e.g., a village or town) with similar areas based on socio-economic indicators and cluster randomise treatments. Again, this was demonstrated in several of the reviewed studies [46,53,83].

Twenty-one of the 47 papers did not include a control group. These were typically pre–post studies, but they were often not paired within subject designs. Multiple studies assessed some of the cohort before the intervention and some of them afterwards, suggesting that the before group acted as a control [38,66]. However, this is not a true control group. Similar strategies involved comparative cross-sectional studies, e.g., [41,46,72,96] where a single cross-sectional survey was conducted and the responses of people who were exposed to the intervention were compared to those that were not. In this way, McDonald and Clements [41] compared survey respondents based on whether they were ‘aware’ of the Bulwell Cat Watch program (community intervention) or not. This type of evaluation does not account for the efficacy of the intervention in being effectively memorable; those who were not aware may have been exposed to the intervention but cannot remember it, particularly because the survey was conducted 2 years after the program began. While cross-sectional studies are less resource intense and can play a valuable role in providing preliminary indications of efficacy, there is a real need within this field to invest in randomised controlled trials.

Besides the lack of controlled trials, there were other methodological issues that warrant noting. The education program under evaluation in Amparo et al. [97] occurred at the same time as a large community program, mass dog vaccination program, and media campaign. Consequently, there is no way of separating the impact of the education component from that of the community-based programs. March et al. [86] included all farmer participants in the stable school intervention, but then those who implemented the recommended measures discussed were considered the ‘treatment’ group and those that did not were considered the ‘control’ group. As such, this study did not test the effectiveness of the intervention to bring about behavioural change, it tested whether the behaviours led to animal health gains.

Another design challenge that should be taken into account is that the use of monitoring logs or technology in control groups may in itself prompt behaviour change. This was evident in Byers et al. [43], where all dog owners in both the control and treatment groups were provided with pedometers and asked to keep a daily log of the time spent in physical activity with their dog. The study identified a similar reduction in dog weight and body condition score in both the treatment and control group, suggesting that the recording techniques may have influenced participants behaviour. Similar effects may arise from control groups simply being aware of the aims of the project and as such, care must be taken in the recruitment process and subsequent communication to not induce expectancy effects.

While RCTs and variations of RCTs comprise the ‘gold standard’, it is possible, and even desirable, to augment the results from such studies with cases studies and correlational data derived from community surveys. It is nevertheless true that pre–post studies with no control group, purely observational studies, or opportunistic quasi-experimental studies are not a substitute for a well-designed RCT.

### 6.6. Evaluation

#### 6.6.1. Outcome Evaluation

Overall, evaluation was often inconsistent and incomplete. As mentioned previously, in order to demonstrate causal pathways between antecedents, behaviour, and animal outcomes, all levels must be evaluated. This was rarely the case in the studies reviewed (Table A3).

Interim outcomes were the most commonly evaluated, presumably because they can be easily measured using a questionnaire. However, many studies did not use any additional measures, thereby relying on the assumption of a causal relationship between the antecedents and behaviour, yet not recognising or discussing this assumption. One exception was Baatz et al. [66] who clearly articulated their strategy of evaluating interim outcomes (attitude change) in their model of change, acknowledged the assumptions, and identified the further work required to evaluate behaviour change; ‘Changes in these [interim] outcomes are assumed to create conditions necessary to cultivate subsequent long-term behaviour change conducive with both safer behaviours when around dogs with the associated potential to prevent dog bites and subsequent dog destructions, and behaviour supportive of more responsible dog ownership.’ (p. 3). In this way, while the basic premise of a behaviour change intervention is to change human behaviour, many of the studies reviewed did not actually evaluate behaviour. Gaafar and Fahmy [40] aimed to evaluate the effect of a training course ‘on the practice and attitudes of the Egyptian researchers who participated.’ (p. 712), yet no measures of behaviour/practice were included. Kanda et al. [71] aimed to ‘improve practices on rabies prevention and pet care’ (p. 349) but used no measures to evaluate pet care nor did they specify what elements of pet care were targeted. Machila et al. [53] aimed to raise ‘the skills and abilities of farmers to identify bovine trypanosomiasis and the appropriate control methods’ (p. 263), but did not evaluate behavioural outcomes, only knowledge. Consequently, it is unknown whether these interventions were actually successful and whether the hypothesised relationships between the antecedents and behaviour were supported empirically. A good example of why this is important is the “The Blue Dog” interactive CD. While both Meints and De Keuster [60] and Schwebel et al. [61] found that the intervention had a positive impact on children’s dog-safety knowledge, Schwebel et al. [61] demonstrated that this did not translate to improved safe behaviour around dogs. This highlights the importance of evaluating behaviour itself in addition to hypothesised causal antecedents. It demonstrates that for this scenario, knowledge may not be sufficient for behavioural change. Alternatively, perhaps the young target audience (3–6 years old) was not able to generalise the lessons from the intervention format (cartoon interactive CD) to real world scenarios. Many of these dog bite prevention programs do not incorporate behavioural measures and as such, could be being delivered at considerable cost, yet having little impact on behaviour.

Where behavioural outcomes were evaluated, this was often through self-reports in questionnaires. While this is a good way to include some behavioural measures when resources do not allow for more direct measurement, they are subject to various reporting biases such as social desirability bias. However, there were several good examples of direct evaluation of behaviour. Approximately a week after delivering a dog safety intervention to school children, Chapman et al. [90] set up a behavioural test in the schoolyard. They tied a Labrador up in the school grounds with its owner 5 m away, disguised as a tradesperson. Childrens’ interactions with the dog were video-taped (without the childrens’ knowledge) and subsequently scored for breaches of the behavioural guidelines taught. Similarly, Morrongiello et al. [59] used a set up situation with a dog in the laboratory where the intervention was being delivered and parent’s supervisory behaviours were recorded.

Despite being the primary target and outcome of behaviour change interventions, human behaviour change is typically an intermediate step to achieving improved animal welfare, behaviour, or productivity. Consequently, it is important to evaluate these animal outcomes to ensure the intervention and behaviours changed are having the intended effect. Only 11 of the 47 papers included direct assessment of animal-based measures. While this is understandable given the resources and multidisciplinary approach required to evaluate both human and animal outcomes, this is a serious inadequacy of current evaluation efforts. An example of why it is important to evaluate animal outcomes and their relationship with human behaviour is Byers et al. [43]. In this study, the proposed relationship between the targeted human behaviour (increased exercise) and the animal outcomes (dog weight loss) were not supported by the data, yet this was not recognised or discussed in the paper. While they instructed dog owners in the treatment group to increase their physical activity with their dogs to 30 min every day, the pedometer data showed no statistically significant change in activity from baseline. However, they still saw significant animal outcomes; dogs in both the control and treatment conditions had a reduction in their weight and body condition scores. All participants had been given a leaflet on preventative health including nutrition. Given that physical activity did not increase (the target behaviour), perhaps the owners were restricting the dog’s feed intake, or just including the dog in their existing physical activities more, though this was not measured (or mentioned). Therefore, it is unknown what behaviours of the owner contributed to this effect and consequently, what future intervention designs should incorporate from the study.

In some instances, tertiary outcomes are more relevant to the aims of the project than individual outcomes and can assist in demonstrating the benefit of behaviour change programs on a broader scale. Few studies included such outcomes in the current review, despite many including them in their aims. While this is not necessarily a failing of individual studies, as many could be considered ‘proof of concept’ type experiments with the purpose of demonstrating that behaviour change is possible, additional work is required to monitor their impact on a larger scale. For example, with many studies focusing on dog bite prevention interventions, longitudinal studies to evaluate their impact on dog bite prevalence (as Deray et al. [63] did) would be beneficial. Bright and Hadden [67] provide another good example of using tertiary outcomes in the evaluation of their training program for shelter volunteers. The study clearly identified that ‘the ideal outcome would be that adoption rates would increase as volunteer and dog behaviour changed for the better’ (p. 97). Tertiary outcome evaluation included the monitoring of adoption rates and the length of stay prior to adoption for 3 years prior to the training implementation and 4 years post. They demonstrated a range of positive changes in dog length of stay and ultimate outcomes (e.g., adoption), thus fulfilling the aim of the project. However, as there was no control group for comparison and dog behaviour and volunteer behaviour were not directly measured (only through volunteer records of ‘risky behaviour’), there is limited evidence that these positive tertiary outcomes were a result of improved human and dog behaviour as suggested in the project aims; there could be other influential factors contributing to the positive result. Hence, the causal pathway and change mechanism was not explicitly tested, making it difficult to determine whether the same approach will be successful elsewhere.

Another important aspect of demonstrating relationships between different outcomes is the use of appropriate statistical methods. Shen et al. [85] used mediation analysis to demonstrate the relationship between knowledge and behavioural simulation. These types of statistical analyses and more complex structural equation modelling were uncommon in the papers found, and should be employed more often.

#### 6.6.2. Process Evaluation

There were a range of intervention context aspects that were evaluated in the studies reviewed. Most commonly, characteristics of the participants, such as age or gender, were evaluated to determine whether the intervention had differing effects. Stringer et al. [35] found that different age groups responded differently to different intervention delivery methods. The knowledge of older participants showed more improvement with the village meeting intervention, whereas younger people improved more with a handout. Rayner et al. [51] investigated differences in learning outcomes between Indian and non-Indian veterinarians (the study was conducted in India). Indian participants had lower baseline knowledge than non-Indian participants, but improved more post-intervention. Meints and De Keuster [60] found that older children improved more than younger children, Tardif-Williams and Bosacki [69] identified both gender and age effects, and Coleman et al. [20] investigated the effect of class size on learning outcomes (though no differences were found).

Other context-related aspects evaluated included the setting and mode of delivery. Coleman et al. [49] aimed to determine whether a cognitive–behavioural intervention that had shown efficacy in a small farm-holder environment [50] would demonstrate similar efficacy in large commercial farms in which stockpersons work in groups and are subject to additional factors such as peer pressure. Descovich et al. [38] compared outcomes of trainees whose trainer had attended in-person or remote training; indeed the in-person training group demonstrated an improvement in knowledge scores, while the remote training group did not. They also demonstrated good recognition and evaluation of other individual factors that impacted program outcomes including the participant’s living area, education, and gender.

Several studies also incorporated some method of evaluating the experience of participants including the acceptability, feasibility, benefits, barriers, and improvements. Utami et al. [47] used a participatory evaluation event where the successes and challenges of the program were prioritised based on a plenary discussion and participatory voting of the entire implementation team. Amparo et al. [97] conducted interviews with teachers delivering the school-based intervention. Importantly, they found compromised fidelity of the program in that teachers did not always follow the lesson guides and flow of activities exactly as suggested in the manual. McDonald and Clements [41] evaluated the acceptability of the program within the community and individual benefits for people actively engaging with the program. Dixon et al. [98] surveyed parents about whether their strategy of delivering a dog bite prevention video in the paediatric emergency department was acceptable and appropriate; 93% of parents supported the strategy. These sorts of evaluations are important as an effective intervention is no use if the target audience will not accept it.

#### 6.6.3. Timing

Timing of evaluation was variable and at times unclear (Table A3). While many only included immediate or short-term evaluation of outcomes, particularly when assessing interim outcomes via questionnaires, a considerable number incorporated longer-term timelines from months to years. While this requires significant resources, it is important to ensure changes are maintained over time. Coleman et al. [20] provides a useful example of why this is important. With testing points at 2 weeks, 2 months, and 4 months post-intervention, they found that while there were positive immediate effects, longer-term retention was low, suggesting that follow-up sessions would be required to maintain behavioural changes.

## 7. Conclusions

The present review has identified little congruence in the design, evaluation, and reporting of behaviour change interventions in animal care and interactive settings. While there has been some promising and important work already conducted, improvement within the design and evaluation elements reviewed here will be critical for the field to progress in a unified way. This can be achieved by following the Ten-Task guide [9]. The main difference for the application of this framework to animal-related settings is that the outcome of interest is often not human behaviour, but the welfare, behaviour, or productivity of the animal. As such, a gold-standard approach would involve directly assessing animal outcomes. However, it is vital to also evaluate behavioural antecedents and behaviour itself in order to demonstrate causal relationships and validate the mechanisms of change. In addition, more standardised and transparent reporting, particularly of intervention content, is required.

**Figure 4:**
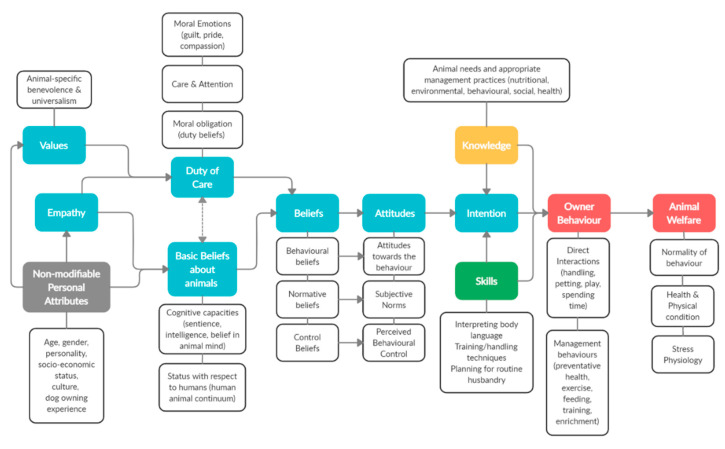
The Pet Care Competency Model: an example of a domain-specific theoretical model, adapted from [99].

An effective way of addressing several of these issues is to develop and report a domain-specific theoretical model that, based on elicitation research, illustrates the hypothesised change mechanisms for the specific issue and context. An example of this is the Pet Care Competency model (Figure 4) [99]. While this is yet to be fully tested empirically, it draws on several established psychological models to present a bespoke map of the proposed elements affecting dog owner behaviour and dog welfare. Consequently, it provides a testable set of mechanisms that can then be clearly mapped to change techniques likely to influence them. This allows for structured and clear design, evaluation, and reporting. For each intervention, it is important to undertake an in-context analysis of what exactly needs to change and how it can be changed, as these are likely to differ across contexts and behaviours. This provides the basis for a comprehensive model of the intervention specifying change mechanisms, contexts, and outcomes.

From the present review and drawing on insights from more established fields of behaviour change, we have compiled a list of specific recommendations to improve the field of human behaviour change research in animal-related settings (Table 3).

Given the influence of human behaviour on the animals we keep and interact with, this is a vitally important field that requires significant work. Adopting these recommendations will assist in developing a rigorous and cohesive science of behaviour change in animal-related settings that can be built upon and progressed. In doing so, this work has the potential to improve outcomes for humans and animals alike.

## Figures and Tables

**Table 1:** Design approaches relevant to animal welfare behaviour change research.

Design	Strengths	Weaknesses	Generalisability
Fully randomised	Strong outcome evidence	Costly in resources	Depends on sampling, attrition
Randomised block	Strong outcome evidence	Require extensive matching	Good if blocks are random
Randomised preference	Strong outcome evidence	Requires careful screening	Good if preferences are random
Nested	Strong outcome evidence	Risk of selection biases	Good if number of groups is sufficient and randomly assigned or matched
Pre–post	Some outcome evidence	No control	Poor
Observational	Good face validity	Mainly descriptive	Poor
Case studies	Good face validity	Poor generalisability	Poor

**Table 2:** Examples and classification of change techniques identified in 47 behaviour change interventions targeting animal care or interactive behaviours, mapped to mechanisms of change and theoretical frameworks.

Examples of Intervention Components (With Study References)	Mechanisms of Change (Theoretical Frameworks)	Change Technique Type/Category
Lectures/information sessions on animal behaviour [48,52] and nutrition [44] Information on animal needs [52,66,69,70], animal husbandry [34,35,41,54,69] and animal disease [43,44,53,54,83] Information on animal welfare issues [38,51], Five Freedoms, and definitions of cruelty [69,84] Leaflet on common animal diseases and prevention measures [43]	Increasing knowledge and changing beliefs (TPB, SCogT, IMB)	Provision of background information
Presenting evidence of the relationship between attitudes and behaviour [49,50,57]	Explaining underlying change mechanisms to participants (TPB, SCogT)	K34 Information about antecedents
Information about what circumstances led to the dog bite incidents [85] Information on production/financial gains/losses of adopting the recommended behaviours [36,49,50,57] Showing video footage of how aversive handling techniques impact ease of handling and production [49,50,57] Highlighting the benefits of a stable, neutered cat population and how trap–neuter–release programs can achieve this [41] explained in [65] Video demonstrating safe and unsafe outcomes of interactive behaviours with dogs [59,60,61] Veterinarian reviewed benefits of owners engaging in physical activity with their dog (unclear whether benefits were for human or dog) [43] Video footage of appropriate and inappropriate handling behaviours and accompanying behavioural responses by animal [49,50,57] Real examples of what happened to the dog following a dog bite incident [85] Real examples of severity of dog bite injury and how it was treated [85]	Explain the consequences that follow from particular actions, including severe consequences. Can change attitudes (TPB, SCogT, IMB)	AM1 Provide information about behaviour–health link AM2 Provide information on consequences (also K35 K36) Fear appeals (emphasising susceptibility to severe consequences) Provide information on consequences for animals
Putting themselves in animals’ shoes (how would you feel) [66] Through dramatization, children explore the idea that animals have feelings [84] Associate the needs of animals with their own needs [70] Role playing and imagination exercises to build empathy [70]	Highlighting how animals may experience the consequence of carers’ actions and that they have emotional responses	K44 Empathy training Animal empathy training
Presented local industry examples of successful implementation of behaviour (stunning standards) [38]	Provide examples of others’ successful performance allowing vicarious learning and prompting changes in normative beliefs (SComT, TPB, SCogT)	AM19 Provide opportunities for social comparison AM3 Provide information about others’ approval (also K38)
Advice to spend at least 30 min a day engaged in physical activity with their dog [43] Develop a class charter as guidance for students ‘to help their animals be healthy and happy [84]	Intention formation and goal setting (CT, TPB, SCogT)	AM4 Prompt Intention formation AM4 Prompt specific goal setting (also K5) K13 Public commitment AM16 Agree on behavioural contract (also K14)
Veterinarian reviewed barriers to physical activity (with dog) [43] Stable schools/farmer field schools: farmers present a problem and collectively identify potential solutions for implementation [37,86,87,88] One-on-one discussion of problems in achieving or maintaining behavioural change [54,57] Presentation of case studies with group discussion of decision-making process [51] Development of individual farm and herd strategies to reduce antimicrobial use [88] Discussion of behavioural strategy to adopt specified animal care tasks [36] Develop an action plan for treatment and control of mastitis [83] Six steps to sound sheep [36] Individualised diet plan [44] or exercise plan [43] for obese dogs Advised to work with their dogs on the class exercises for 15–20 min/day [48]	Anticipation and specification of barriers to goal achievement (CT, SCogT) Formulating specific action plans to overcome identified barriers (CT, SCogT)	AM 5 Prompt barrier identification K7 Problem solving AM 10 Prompt specific goal setting K8 Action planning
Instruction on how to interpret dog body language [20,48,52,66,67,89,90,91,92] Instruction on how to read dog food labels [44] Instruction on safe behaviour around dogs [20,66,90,92,93] Instruction on how to train animal using positive reinforcement [48,67,69] Instruction on how to recognise clinical signs of mastitis [83]	Provision of instruction and or training to increase skills (IMB, CT, SCogT)	AM 8 Provide instruction (also K56)
Videos of appropriate and inappropriate handling behaviour [49,50,57,59,60,61] Demonstrating ‘do’s’ and ‘don’ts’ of behaviour around dogs [90] Demonstrate how to build model pig pen [54]	Modelling or demonstrating a new behaviour to allow modelling/imitation to develop skills (IMB, SCogT)	AM9 Model/ demonstrate the behaviour
Verbal feedback incorporated into computer self-test, e.g., “Well done! You let the dog rest. That was the right choice!” [60]	Receiving feedback on decision making and/or action (CT, OC)	AM13 Provide feedback on performance (also K15)
Evaluation of herd health status provided to farmers [86] GPS tracking of pet cats and demonstration of where they roam [42] Monthly weigh ins for obese dogs [44]	Receiving feedback on the outcome of behaviour (CT, OC)	K16 Feedback on outcome(s) of behaviour
Posters in workplace [50,57]. Newsletters summarising intervention messages [50,57,70] Follow-up letter summarising discussion with detailed flock specific advice [36] Follow-up visit to emphasise message [54,57] Take-home materials for parents/guardians for reinforcement of messages [92,93]	Provide environmental cues to prompt goals/ planning action (OC, CT)	AM18 Use follow-up prompts (also K60)
Practice (during the class) different steps regarding how to approach and handle dogs safely [20,84,90] Role play of appropriate behaviour with an imaginary angry dog [20] or dog toys [93] or puppets [92]	Facilitating practice of new behaviour to develop skills and routinise behaviour to initiate habit formation (IMB, CEOS)	AM17 Prompt practice (also K69) K70 Habit formation
Prompting self-reflection and evaluation of whether they had changed their behaviour [50,57]	Prompting recipients to reflect on their goal achievement/ behaviour change (CT)	AM11 Prompt review of behavioural goals
Identifying situations where negative handling may be prompted or considered necessary, but demonstrating that this is avoidable [50,57]	Anticipating future situations in which plans may not be achievable and cognitive rehearsal of how to manage these situations (CT)	AM 23 relapse prevention Inoculation through preparation for challenging circumstances
Trap–neuter–release program for stray cats [41] Community health days—free preventative veterinary care [47] Provision of trained community-based agents to support community members with troubleshooting dog-related concerns [47]	Provision of practical services and support for change (CT, SCogT)	AM20 Plan social support/social change K54 Obtain practical social support Community service provision

AM = technique categorised from Abraham and Michie Taxonomy [6]; K = from Knittle et al., compendium [21].

**Table 3:** Recommendations to improve research into behaviour change interventions in animal care and interactive settings.

Action *Relevant Ten-Task Guide Task*	Sub-Actions
Conduct elicitation research *Tasks 1–3*	Identify specific target behaviours Use theoretical models of behaviour to investigate a range of behavioural antecedents and identify those most influential
Construct a domain-specific hypothesised model *Task 4*	Draw on psychological models to clearly articulate the hypothesised change mechanism(s) for the specific behaviours of interest
Map change techniques to mechanisms *Task 5*	Consider the full range of available, tested techniques Select techniques that map to the identified change mechanism(s)
Co-design and pre-test interventions *Tasks 5–7*	Involve end users in the design process to refine delivery modes and ensure comprehension, acceptability, feasibility
Conduct randomised controlled trials where possible *Task 8*	Ensure participants are randomly assigned to control or treatment groupsWhere RCT is not possible, at least include a control group Consider sampling biases when generalising to populations
Conduct evaluation that matches the aims and change mechanisms *Task 9*	Measure interim (antecedents), primary (behaviour), and secondary (animal) outcomes Select appropriate time points to measure endurance of change over time
Clearly report all aspects	Include intervention manuals in publications Use appropriate language to clearly describe change techniques (e.g., from existing taxonomies) Include models, tables, and timelines clearly demonstrating how the intervention maps to techniques, mechanisms of change, and evaluation measures

## Appendix A

**Table A1:** Research design elements.

Paper	Topic	Target Audience	Research/Experimental Design Elements	Control Group	Theoretical Framework
Amparo et al. [97]	Dog bite/rabies prevention	Children (5–14 yo)	Pre–post	N	No
Arbour et al. [84]	Companion animal treatment	Children (9 yo)	Quasi-experimental; pre–post (paired within subject), unknown whether randomised (only two classes)	Y	No
Auplish et al. [89]	Dog bite/rabies prevention	Children/Teenagers (10–17 yo)	Pre–post	N	No
Baatz et al. [66]	Dog bite prevention and dog welfare	Children (7–11 yo)	Comparative cross-sectional (pre vs. post), random allocation to pre vs. post	N	Theory of Change
Bright and Hadden [67]	Shelter volunteer training—dog interaction	Adult	Pre–post	N	Model of Instructional Design
Byers et al. [43]	Dog obesity	Adult	RCT with pre–post measurements	Y	No
Chapman et al. [90]	Dog bite prevention	Children (7–8 yo)	RCT, cluster randomisation	Y	No
Chilundo et al. [54]	Animal agriculture—pigs	Adult	Quasi-experimental; allocation not random	Y	No
Clark and Boyer [48]	Companion dog behaviour/relationship	Adult	RCT with pre–post measurements	Y	No
Coleman et al. [20]	Dog bite prevention	Children (5–6 yo)	RCT, 2 × 2 factorial design	Y	No
Coleman et al. [49]	Animal agriculture—pigs	Adult	Quasi-experimental; allocation not random	Y	No
da Cunha et al. [96]	Companion animal management and welfare	Children (8–14 yo)	Comparative cross-sectional	N	No
Deray et al. [63]	Rabies prevention	Children (5–14 yo)	Pre–post	N	No
Descovich et al. [38]	Animal agriculture—abattoir	Adult	Comparative cross-sectional, (pre training vs. post-training)	N	No—though did mention TPB in introduction and some questions seem to reflect TPB
Dias Costa et al. [45]	Companion animal management and population control	Adult	Pre–post (matched within subject)	N	No
Dixon et al. [98]	Dog bite prevention	Children (5–9 yo)	Pre–post	N	No
Franco and Olsson [39]	Animals in research	Adults	Pre–post	N	No
Gaafar and Fahmy [40]	Animals in research	Adults	Pre–post	N	No
Grant et al. [36]	Animal agriculture—sheep	Adults	Quasi-experimental; pre–post, no random allocation of main treatments (delivery mode) but random allocation to pos/neg framed messaging within one of the treatment arms	Y	No
Hasanov et al. [46]	Rabies prevention	Adults	Comparative cross-sectional	Y	No
Hemsworth et al. [50]	Animal agriculture—pigs	Adults	RCT with pre–post measurements	Y	No
Hemsworth et al. [57]	Animal agriculture—dairy cows	Adults	RCT with pre–post measurements	Y	No
Henriksen et al. [37]	Animal agriculture—mink	Adults	Qualitative post-interviews	N	No
Kanda et al. [71]	Rabies prevention	Children (grade 5)	Quasi-experimental; pre–post (within subject), allocation not random	Y	No
Karimuribo et al. [83]	Animal agriculture—dairy cows	Adults	RCT cluster randomisation (village), with pre–post measurements	Y	No
Khode et al. [72]	Animal agriculture—dairy cows	Adults	Comparative cross-sectional (post), propensity score matched control group	Y	No
Lakestani and Donaldson [91]	Dog bite prevention	Children (3–5 yo)	Quasi-experimental; allocation not random (non-equivalent control group design), pre–post	Y	No
Machila et al. [53]	Animal agriculture—cattle	AdultsChildren	Quasi-experimental; allocation not random, pre–post for school children, post only for farmers	Y	No
March et al. [86]	Animal agriculture—dairy cows	Adults	Pre–post	N	No
Mariti et al. [52]	Companion animal management and welfare	Children (9–11 yo)	Pre–post (repeated measures)	N	No
McDonald and Clements [41]	Cat overpopulation (TNR)	Adults	(1) Comparative cross-sectional (2) Pre–post	N	COM-B/Behaviour Change Wheel
Meints and De Keuster [60]	Dog bite prevention	Children	Pre–post, 2 treatment groups (randomly assigned)	N	No
Morrongiello et al. [59]	Dog bite prevention	Adults	RCT with pre–post measurements	Y	No
Nicoll et al. [70]	Companion animals and welfare	Children (first grade)	RCT cluster randomised (class), 2 × 2 factorial design, with pre–post measurements	Y	No
Rayner et al. [51]	Veterinarians—animal welfare, animal ethics, euthanasia	Adult	Quasi-experimental; allocation not random, pre–post measurements	Y	No
Roetman et al. [42]	Cat containment	Adult	Quasi-experimental; allocation not random, pre–post measurements (paired)	Y	No
Schwebel et al. [61]	Dog bite prevention	Children (3.5–6 yo)	RCT with pre–post measurements	Y	No
Shen et al. [85]	Dog bite prevention	Children (9–11 yo)	RCT with pre–post measurements	Y	No—mentions HBM in intro
Spiegel [93]	Dog bite prevention	Children (5–12 yo)	Pre–post	N	No
Stringer et al. [34]	Working donkey health	Adult	RCT, cluster randomised (village)	Y	Participatory Situation Analysis
Stringer et al. [35]	Working donkey health	Adult	RCT, cluster randomised (village)	Y	Participatory Situation Analysis
Tardif-Williams and Bosacki [69]	Companion animals and welfare	Children (6–12 yo)	Pre–post (paired within subject)	N	No
Utami et al. [47]	Dog welfare and rabies prevention	Adult	Pre–post (paired within subject)	N	Theory of Change
Vaarst et al. [88]	Animal agriculture—dairy cows and antibiotics	Adult	Pre–post	N	No
Vaarst et al. [87]	Animal agriculture—dairy cows	Adult	(1) Pre–post (2) Cross-sectional	N	No
Wilson et al. [92]	Dog bite prevention	Children (4–6 yo)	RCT cluster randomised (class), 2 × 2 factorial design, with pre–post measurements	Y	No
Yaissle et al. [44]	Dog obesity	Adult	RCT with pre–post measurements	Y	No

**Table A2:** Intervention targets and content.

Paper	Intervention Targets	Outcome Behaviour Clearly Defined	Secondary Outcome (Animal)	Tertiary Outcome	Intervention Format	BCTs Able to Be Coded
Amparo et al. [97]	Knowledge	No—vague; ‘animal bite prevention, bite management, and responsible pet ownership’	NS	Reduced dog bite incidence	Curriculum manual for school teachers	No
Arbour et al. [84]	Empathy Knowledge Behaviour	No—‘humane behaviour’ though some able to be deduced from eval.	NS	Human-directed empathy	8 × 1 h sessions in school delivered by teacher (last session field trip to RSPCA)	Some
Auplish et al. [89]	Knowledge Skills	No—ability to interpret dog body language, but does not specify actions	NS	Reduced dog bite incidence	45 min education session	Some
Baatz et al. [66]	Knowledge Attitudes	No—vague; “safe behaviour” “and “responsible dog ownership”	Improve canine welfare	Reduced euthanasia	1 × 1 h session with Dogs Trust education officer at school	Yes
Bright and Hadden [67]	Knowledge Attitudes Skills Behaviour	Yes—broad category, but many specific behaviours also defined	Improved dog behaviour	Increased adoption rates	Multilevel training course combining Ppt presentations, hands on demonstration/practice, and tests	Some
Byers et al. [43]	Behaviour	Yes—spend at least 30 min a day engaged in physical activity with dog	Improved body condition (lose weight) and biochemical measures of health	NS	Veterinary counselling session (10 min)	Yes
Chapman et al. [90]	Skills Behaviour	Yes—various safe behaviours explicitly described	NS	Reduced dog bite incidence	1 × 30 min lesson with an ‘accredited’ dog handler at school and resource kit for teachers	Some
Chilundo et al. [54]	Knowledge Behaviour	Yes—clear table with outcome variables	Pig Productivity	NS	1.5 day village pig farming education session + second session 9 months later	Some
Clark and Boyer [48]	Knowledge Human and Dog Behaviour	Yes—spending 20 min/day with dog, attend obedience classes	Improved dog behaviour	Improved human–animal relationship	Treatment 1: weekly obedience classes Treatment 2: spend 20 min with dog daily	Some
Coleman et al. [20]	Knowledge Skills Behaviour	Yes—detailed description of what constitutes ‘correct’ responses to dog	NS	Reduced dog bite incidence	30 min education session (with live dog)	Some
Coleman et al. [49]	Attitudes Behaviour	No—implied through behavioural measurements	Pig behaviour and welfare—withdrawal response	NS	1 h small group cognitive–behavioural intervention	Some
da Cunha et al. [96]	Knowledge	No—vague; ‘responsible pet ownership’	NS	Pet population management, relinquishment prevention, create a more humane and pet-friendly community.	4 year outreach educational program integrated into school curriculum (delivered by teachers) + a competition	No
Deray et al. [63]	Knowledge Resources	No—vague; dog bite prevention/management and responsible pet ownership.	NS	Reduced dog bites and rabies incidence	Multifaceted community program, education integrated into school curriculum + vaccination	No
Descovich et al. [38]	Knowledge Attitudes	No—vague; improved handling and stunning	Improved animal welfare	NS	Workshop Posted training materials with instructions	Few
Dias Costa et al. [45]	Behaviour—Unclear	No—some behaviours implied through evaluation measures	NS	Animal population management	3 year community program—not described	No
Dixon et al. [98]	Knowledge	No—vague; safe behaviour around dogs, can deduce from evaluation would include when to safely approach	NS	Reduced dog bite incidence	20 min video on how to be safe around dogs; “Dogs, Cats and Kids”	No
Franco and Olsson [39]	Attitudes Behaviour	No—vague; applying the 3Rs	NS	Responsible use of animals in research	FELASA professional course for researchers	No
Gaafar and Fahmy [40]	Knowledge	No—vague; animal care and use, ethical consideration, 3Rs	NS	Standardisation of animal use	2 day professional training course	No
Grant et al. [36]	Attitudes Behaviour	Yes—clearly defines 6 behaviours	NS	NS	3 intervention types: postal, group, one on one	Yes
Hasanov et al. [46]	Knowledge Awareness Behaviour	No—deduced from evaluation, dog vaccination	Dogs vaccinated	Reduce human cases of rabies and improve surveillance	Posters, leaflets and text messages.	No
Hemsworth et al. [50]	Attitudes Behaviour	Partly—‘positive handling behaviour’, then specific behaviours deduced from evaluation measures	Improve behaviour, welfare, and productivity of pigs	NS	1 h one-on-one cognitive–behavioural intervention + reinforcing posters and newsletters.	Yes
Hemsworth et al. [57]	Attitudes Behaviour	Partly—‘positive handling behaviour’, then specific behaviours deduced from evaluation measures	Improve behaviour and productivity of dairy cows	NS	1 h one-on-one cognitive–behavioural intervention + reinforcing posters, newsletters, and follow-up session	Yes
Henriksen et al. [37]	Knowledge	No—vague; ‘improve management’	Improve mink welfare	NS	“Stable schools” 5 sessions over one year.	Few
Kanda et al. [71]	Knowledge	No—though some could be inferred from questionnaire	NS	Reduce human rabies cases	‘Rabies Edutainment 4 Kids’: lessons integrated into school curriculum + leaflet	No
Karimuribo et al. [83]	Knowledge	No—treatment and control of mastitis but no specific actions	NS	Reduce mastitis prevalence	Indirect: Mastitis training course + handouts, posters, videos, branded pens. Direct: Village meeting + video, handout	Some
Khode et al. [72]	Knowledge	No—vague; ‘scientific dairy practices’	NS	NS	Various existing education courses delivered by Agricultural Extension Centres (Krishi Vigyan Kendras)	No
Lakestani and Donaldson [91]	Knowledge Skills	Partly—’how to behave in the presence of a scared dog’ and not to approach a scared dog.	NS	Reduced dog bite incidence	Video-based intervention at school (facilitated by researcher)	Some
Machila et al. [53]	Knowledge Skills	Yes—appropriate trypanocidal use described	Reduced mortality	Prevent trypanocidal resistance, improve farm productivity	Leaflets and posters	Some
March et al. [86]	Knowledge	No—vague; management behaviour	Improve cow health and welfare	NS	2 years of “stable schools”	Few
Mariti et al. [52]	Knowledge	Partly—‘humaneness’, some deduced from information in sessions (e.g., when/when not to approach a dog)	Unclear	Psychological and physical benefits to child of positive HAR	4 × 40 min in-class sessions by researchers	Few
McDonald and Clements [41]	Knowledge Attitudes	Yes—reporting of unowned cats for neutering	Roaming cats trapped and neutered	Reduced cat population	Multifaceted community outreach program + direct support (TNR)	Some (described in [65])
Meints and De Keuster [60]	Knowledge Skills	Partly—broadly described as safe behaviour around dogs in paper, but specific safe and unsafe behaviours clearly described in the intervention scenarios (supp. materials)	NS	Reduced dog bite incidence	Interactive CD “The Blue Dog”	Yes
Morrongiello et al. [59]	Knowledge	Yes—supervision behaviour inc. attention to child, proximity, and reactions	NS	Reduced dog bite incidence	Interactive CD “The Blue Dog” and accompanying parent manual	Some; CD yes, manual no
Nicoll et al. [70]	Attitudes Empathy	No—topics described but not behaviours	NS	NS	In-class sessions with humane educator and therapy dog; 6 sessions over 4 months + humane education newsletter	Some
Rayner et al. [51]	Knowledge Attitudes	No—learning objectives but no specific behaviours	NS	NS	A lecture and cases studies embedded within a 2 week professional training course (surgical skills)	Few
Roetman et al. [42]	Knowledge Attitudes	Yes—confine cats to property, though also measured some other cat management behaviours which were not outlined in the approach	Cat behaviour- less roaming	Reduced predation, welfare impacts on cats, disease transmission, annoyance	‘Citizen science’ project—GPS tracking of cats	Yes
Schwebel et al. [61]	Knowledge Behaviour	Partly—risky behaviour and some examples given	NS	Reduced dog bite incidence	Interactive CD “The Blue Dog”	Yes
Shen et al. [85]	Knowledge Attitude	No—vague; risky behaviour	NS	Reduced dog bite incidence	Video of dog bite testimonials	Yes
Spiegel [93]	Knowledge Behaviour	Partly; ‘how to avoid and prevent threatening situations that often lead to attacks’	NS	Reduced dog bite incidence	Multiactivity 60 min session; workbook, discussion, role play, video	Some
Stringer et al. [34]	Knowledge	No—some deduction from learning objectives	Reduced donkey wounds	NS	1. Diagrammatic Handout 2. Radio Drama 3. Village meeting	Few
Stringer et al. [35]	Knowledge	No—some deduction from learning objectives	Reduced donkey wounds	NS	1. Diagrammatic handout 2. Radio Drama 3. Village meeting	Few
Tardif-Williams and Bosacki [69]	Knowledge Attitudes Behaviour	No—vague; “promote positive interactions (proper treatment and close emotional bonds)”	NS	Quality of child–companion animal relationships	Five-day humane education summer-camp program at animal shelter	Some
Utami et al. [47]	Knowledge Behaviour Resources	Partly—implied through evaluation; measures vaccination and providing better care for dogs	Improved dog physical condition, vaccination, sterilisation, confinement	Maintain herd immunity and rabies control	Community based multifaceted program	Some
Vaarst et al. [88]	Knowledge Behaviour	No—overall goal defined eliminate antibiotic use, actions required to achieve that not defined	Minimise disease	Eliminate use of antibiotics in organic dairy herds	Monthly “stable schools” for 1 year	Few
Vaarst et al. [87]	Knowledge Behaviour	No—‘animal health promotion with emphasis on mastitis’, specific action not defined	Reduce mastitis incidence/increase productivity	NS	Participatory farmer field schools 2/month for 12 months	Few
Wilson et al. [92]	Knowledge Behaviour	Partly—increased caution approaching dogs	NS	Reduced dog bite incidence	2 elements: (a) 30 min instructional session, (b) parent information package	Some
Yaissle et al. [44]	Knowledge Behaviour	Yes—adhere to individualised diet plan and increase physical activity	Weight loss	NS	Monthly education sessions on nutrition-related topics	Some

NS = not specified in paper.

**Table A3:** Evaluation elements.

Paper	n *	Evaluation Tool	Outcome Evaluation Measures				Process Evaluation	Evaluation Timing
			Interim	Primary	Secondary	Tertiary		
Amparo et al. [97]	365	Pre–post survey (children) Focus group (teachers)	Knowledge questions	-	-	Self-report bite incidents	Focus group (teachers)	1 year post
Arbour et al. [84]	37 (23/**14**)	Pre–post survey	Bryant’s empathy index	Children’s Treatment of Animals Questionnaire (self-report behaviour),	-	-	-	Unclear
Auplish et al. [89]	261	Pre–post survey	Knowledge questions	-	-	-	-	Unclear
Baatz et al. [66]	2732 (post 621/726 **pre 632/753**)	Pre–post survey (between subject)	Attitude questions	-	-	-	-	Immediately after intervention
Bright and Hadden [67]	2000 trainees. 1982 dog records (eval.)	Data from shelter and volunteer records	-	-	-	Adoption rates, euthanasia rates, dog length of stay in shelter, number of ‘risky’ incidents	Number of volunteers trained	3 time periods: (1) 30 months pre, (2) 30 months post, (3) 30–57 months post
Byers et al. [43]	32 (22/**10**)	Veterinary examination (pre–post)Owner survey (pre–post)Pedometer (owner; pre-post)	Owner reported: perceived health, physical activity, social support, stress, strength of human–dog relationship	Owner physical activity (pedometer)	Dog BCS, BMI, serum biochemical marker	-	-	1 week pre 3 months post
Chapman et al. [90]	346 (197/**1****49**)	Behavioural test in set up situation (children unaware)- behaviour video recorded	-	Number of children who breached the proscribed behaviours taught	-	-	-	7–10 days post
Chilundo et al. [54]	90 (49/**41**)	Interview-based Questionnaire Direct assessment of facilities pre and post for both	Knowledge questions	Management/facility measures: type of pig pen, confinement, etc.	Hygiene status	-	-	24 months post first session
Clark and Boyer [48]	30 (assumed 10/10/**10**)	Dog behaviour test, owner questionnaire, daily log sheets of time spent	Profile and the Pet Attachment Survey	-	Proximity to owner, tactile behaviour, separation behaviours, obedience behaviours, owner-reported behaviour issues	-	-	5 separate behaviour test sessions—3 pre and 2 post (concluding 1 week post-intervention)
Coleman et al. [20]	123 (30/30/31/**32**)	Role play, questionnaire, visual matching activity	Knowledge questions	Approach behaviour demonstration	-	-	-	Within 2 weeks post, 2 months post and 4 months post
Coleman et al. [49]	43 (18/**25**)	Pre–post survey, direct observations of handling behaviours, pig behaviour (withdrawal response)	Multiple surveys: (1) General beliefs about pigs, (2) behaviour beliefs, (3) job satisfaction, (4) technical knowledge and willingness to learn	Direct observations of handling behaviours	Pig behaviour (withdrawal response)	-	-	6 months prior for job-related data, 1–3 weeks prior for attitudinal and behavioural data, 6 months post
da Cunha et al. [96]	1332	Student Survey (outcome) Teacher survey (process)	Knowledge questions	Self-reported management	-	-	Teacher survey, Reach	Unclear
Deray et al. [63]	255	Pre–post survey Dog bite incidence	Awareness (measures unclear)	-	-	Dog bite incidence	-	1 month post
Descovich et al. [38]	308 (post 147 pre 161)	Pre–post survey (between subject design)	Knowledge questions Attitudinal questions	-	-	-	-	Unclear
Dias Costa et al. [45]	70	Interview-based questionnaire	attitudes towards stray dogs/population management	Reported management behaviours	-	-	-	3 years between pre and post
Dixon et al. [98]	120	Pre–post questionnaire (children) Post questionnaire (parents)	Children: 14 different scenarios presented and asked what the child would do (yes/no)	-	-	-	Parents: acceptability of intervention	Immediately pre and post
Franco and Olsson [39]	206	Pre–post questionnaire (pooled)	knowledge, attitudes, hypothetical application scenario	-	-	-	-	Immediately pre, 1 year post
Gaafar and Fahmy [40]	100	Pre–post questionnaire	Knowledge about 3Rs	-	-	-	-	Immediately pre and post
Grant et al. [36]	199 (29/51/660/**119**)	Pre–post questionnaire for all Pre–post farm visits for one-on-one group	knowledge, attitudes,	Self-reported behaviour	-	Flock lameness prevalence (self-reported)	-	Varied—immediately to months
Hasanov et al. [46]	672 (336/**336**)	Post survey (comparing intervention to control area)	Self-reported awareness, actual knowledge questions, awareness,	-	Vaccination status of animals	-		Unclear
Hemsworth et al. [50]	25 (13/**12**)	Pre–post questionnaire, Direct observations: human and pig behaviour Pig reproductive data	Attitudes	Direct observations of handling behaviour	Pig behaviour: approach test (fear of humans) Reproductive: piglets born/sow/year, pigs weaned/sow/year	-		8 month pre-intervention period to a 15 month post-intervention period
Hemsworth et al. [57]	Trial a) 29 Trial b) 94	Pre–post questionnaire Direct observations: human and cow behaviour Cow stress physiology and production	Questionnaire: attitudes, working conditions	Direct observations of handling behaviour	Cow behaviour: approach test (fear of humans), behaviour at milking Milk cortisol, milk yield, milk protein, milk fat	-	Questionnaire included perception of intervention	Approximately 1 year post
Henriksen et al. [37]	13	Semi-structured qualitative interviews	-	Self-reported practice changes	-	-	Experiences and improvements	After 1 year of intervention period
Kanda et al. [71]	125 (73/**52**)	Pre–post questionnaire (paired, within subject)	Knowledge questions	-	-	-		4 weeks between pre- and post-survey with the intervention delivered sometime in that 4 week period
Karimuribo et al. [83]	360 (unclear)	Pre–post-interview	Knowledge: ‘volunteering mastitis facts’	-	-	-		1 month post and 16 months post
Khode et al. [72]	360 (90/**270**)	Knowledge questionnaire (post only- compare control with treatment)	Knowledge questions: breeding, feeding, management, health.	-	-	-		Varied—reference period was 3 years prior to survey
Lakestani and Donaldson [91]	70 (36/**34**)	Identifying dog behaviour/emotions from video clips	“How is this dog feeling? Is it happy, scared or angry?” “How do you know it’s feeling that way?”	-	-	-		2–3 days pre, 2–3 days post
Machila et al. [53]	558 (194/**364**) school children 267 (125/**142**) farmers	Interview based questionnaire (pre–post for school children, post only for farmers)	Knowledge questions—signs of disease and appropriate treatment	-	-	-	Exposure/reach	4–6 weeks post
March et al. [86]	19	Pre–post on-farm assessment Farmer interviews	-	-	Cow BCS, locomotion, cleanliness, injuries,	Herd average milk production, disease treatments/cow/year	Farmer: perceptions of the stable school, benefits to participation, improvements	2 years baseline data, 2 year intervention period
Mariti et al. [52]	201	Pre–post questionnaire	knowledge of animals, perception of non-human animals, responsibility toward pet species	-	-	-		Immediately pre and post
McDonald and Clements [41]	478 (cross sectional) 21 (pre post) 34 (targeted survey)	Cross-sectional survey Pre–post survey Targeted survey of participants	Knowledge, behavioural intentions, self-efficacy	Self-reported behaviour	-	-	Free answer in cross-sectional survey Targeted survey—benefits and outcomes of participating	2 years post-launch of community program
Meints and De Keuster [60]	96	Pre–post scenarios with behavioural choices	8 scenarios presented, child had to choose the safe behaviour	-	-	-		Immediately pre, immediately post, 2 weeks post
Morrongiello et al. [59]	55 (27/**28**)	Video-taped behavioural observations (set up situation with dog; participant unaware)	-	Caregiver’s supervisory behaviours (attention to the child, proximity, reactions)	-	-		3 week interval between pre and post
Nicoll et al. [70]	148 with pets: (26/29/29/**24**)	Pre–post questionnaire	Primary Attitude Scale	Companion Animal Bonding Scale (inc. self-reported behaviour/interactions)	-	-		Immediately before, ~6 weeks post
Rayner et al. [51]	148 (84/49/**15**)	Pre–post questionnaire	Knowledge questions, attitude questions	-	-	-		Immediately after intervention
Roetman et al. [42]	410 (varied with analysis)	Pre–post questionnaire (within subject)	attitude questions, knowledge questions about how far their cat travels	Self-report behaviour	-	-		Unclear
Schwebel et al. [61]	76 (37/**39**)	Pre–post knowledge and behaviour tests (outcome) Parent survey (process)	(a) recognition of safe/risky behaviour from pictures (b)	(a) Acting out safe behaviour with dollhouse (b) Video-recorded behaviour with unfamiliar live dog.	-	-		3 week interval between pre and post
Shen et al. [85]	276 (141/**135**)	Pre–post (1) questionnaire (2) dollhouse behaviour simulation	Questionnaire: knowledge about interacting appropriately with dogs, and perceived vulnerability to dog bites	Dollhouse simulation: risky behaviour with dogs	-	-		1 week pre, 1 week intervention, 1 week post
Spiegel [93]	486	Pre–post questionnaire	knowledge of dog behaviour, body language, and how to prevent dog bites	Self-reported behaviour	-	-	Use and enjoyment of the program	2 weeks pre, 2 weeks post
Stringer et al. [34]	504 (114,124,137,**129**)	Pre–post questionnaires	Knowledge questions	-	-	-	-	10–18 days post
Stringer et al. [35]	476 (109/128/114/**125**)	Pre–post questionnaires	Knowledge questions	-	-	-	-	6 months post
Tardif-Williams and Bosacki [69]	77	Pre–post questionnaires	Pet Friendship Scale, Comfort from Companion Animal Scale and a drawing task	Self-reported: Companion Animal Bonding Scale and Children’s Treatment of Animal Questionnaire	-	-	-	Immediately pre and post
Utami et al. [47]	2098 (dogs)	In-home visit with pre–post direct observations (dog welfare), questionnaire (interview based), and street surveys (roaming dog density)		Dog confinement practices (self-reported)	Body condition score, skin conditions, injuries	Vaccination coverage, sterilisation status, roaming dog density	-	Varied
Vaarst et al. [88]	23	Pre–post qualitative interviews Post focus groups	Perceptions of changes on farm	Self-reported changes to practice	-	-	Experience with participation	1 year between baseline and post-evaluation
Vaarst et al. [87]	37	Participatory Impact Assessment Evaluation workshops Cow measures	Knowledge (listing what they had learned)	-	Milk production (L/month/cow), teat hygiene,	Mastitis incidence	Experience with participation	2 year follow-up
Wilson et al. [92]	177 (48/54/65/**10**)	Pre–post questionnaire (parents) Pre–post photo activity (children)	Parent questionnaire: beliefs about child’s behaviour with dogs, bite history Photo activity: range of safe and unsafe situations presented in photos—child to respond yes/no to whether they would pat the dog or not	-	-	-	-	Immediately pre, 4 weeks post
Yaissle et al. [44]	32 (16/**16**)	Veterinary exam	-	-	Body condition score, weight	-		Monthly for 24 months (6 months weight-loss period, 18 month maintenance period)

* (treatment(s)/control or comparison group).
